# Glucokinase inhibitor glucosamine stimulates feeding and activates hypothalamic neuropeptide Y and orexin neurons

**DOI:** 10.1016/j.bbr.2011.03.043

**Published:** 2011-09-12

**Authors:** Ligang Zhou, Chen-Yu Yueh, Daniel D. Lam, Jill Shaw, Mayowa Osundiji, Alastair S. Garfield, Mark Evans, Lora K. Heisler

**Affiliations:** a Department of Pharmacology, University of Cambridge, Cambridge, Tennis Court Road, Cambridge CB2 1PD, UK; b Institute of Metabolic Science, Department of Medicine, University of Cambridge, Cambridge CB2 0QQ, UK; c Department of Endocrinology, Shuguang Hospital, Shanghai University of TCM, China; d Department of Family Medicine, Chang Gung Memorial Hospital at Chiayi, Taiwan; Chang Gung Institute of Technology, Taiwan

**Keywords:** Glucosamine, Glucokinase, Food intake, Hypoglycemia, Neuropeptide Y, Orexin, Melanocyte-stimulating hormone, Melanin concentrating hormone, Hypothalamus

## Abstract

Maintaining glucose levels within the appropriate physiological range is necessary for survival. The identification of specific neuronal populations, within discreet brain regions, sensitive to changes in glucose concentration has led to the hypothesis of a central glucose-sensing system capable of directly modulating feeding behaviour. Glucokinase (GK) has been identified as a glucose-sensor responsible for detecting such changes both within the brain and the periphery. We previously reported that antagonism of centrally expressed GK by administration of glucosamine (GSN) was sufficient to induce protective glucoprivic feeding in rats. Here we examine a neurochemical mechanism underlying this effect and report that GSN stimulated food intake is highly correlated with the induction of the neuronal activation marker cFOS within two nuclei with a demonstrated role in central glucose sensing and appetite, the arcuate nucleus of the hypothalamus (ARC) and lateral hypothalamic area (LHA). Furthermore, GSN stimulated cFOS within the ARC was observed in orexigenic neurons expressing the endogenous melanocortin receptor antagonist agouti-related peptide (AgRP) and neuropeptide Y (NPY), but not those expressing the anorectic endogenous melanocortin receptor agonist alpha-melanocyte stimulating hormone (α-MSH). In the LHA, GSN stimulated cFOS was found within arousal and feeding associated orexin/hypocretin (ORX), but not orexigenic melanin-concentrating hormone (MCH) expressing neurons. Our data suggest that GK within these specific feeding and arousal related populations of AgRP/NPY and ORX neurons may play a modulatory role in the sensing of and appetitive response to hypoglycaemia.

## Highlights

► Antagonism of brain glucokinase by glucosamine (GSN) stimulates feeding. ► GSN-stimulated feeding is associated with increased hypothalamic neuronal activity. ► Activity in arcuate and lateral hypothalamus is strongly correlated with GSN-feeding. ► GSN activates orexigenic arcuate neuropeptide Y and lateral hypothalamus orexin cells.

The detection and maintenance of physiologically appropriate levels of glucose is paramount to mammalian viability. Multiple physiological systems, therefore, operate to detect fluctuations in glucose levels and to prompt the induction of apposite counter-regulatory responses, including ingestive behaviour. Glucose, as the primary fuel source in mammals, is a key indicator of nutritional state, with low or falling blood glucose levels triggering defensive physiological responses and hunger. However, for glucose to be able to influence feeding behaviour, whether as an emergency response to glucoprivation or within a more normal appetitive context, the brain must be able to accurately and rapidly detect oscillations in interstitial glucose levels. In this regard, the hypothalamus has been identified as a key component of the centrally regulated energy homeostasis network.

Within the basomedial hypothalamus, the melanocortin system in the arcuate nucleus (ARC) plays a critical role in energy balance; the orexigenic endogenous melanocortin receptor antagonist agouti-related peptide (AgRP) is co-expressed with orexigenic neuropeptide Y (NPY), while the anorectic endogenous melanocortin receptor agonist alpha-melanocyte simulating hormone (α-MSH) is co-expressed with anorectic cocaine and amphetamine regulated transcript (CART) [1]. These basomedial populations may form part of an integrated homeostatic network with neurons of the lateral hypothalamic area (LHA) that express orexin/hypocretin (ORX) or melanin concentrating hormone (MCH). In controlling energy balance, these neurons respond to a complex series of nutritional cues, including those communicated by peripherally derived circulating factors or relayed by visceral nerves. In addition, some neurons are able to influence energy homeostasis in response to directly sensed changes in the levels of specific brain substrates within their local environment [2,3]. In this regard, AgRP/NPY, α-MSH, ORX and MCH neurons sense changes in extracellular glucose concentration [4–8]. Such neurons can exhibit excitatory (glucose-excited, GE) or inhibitory (glucose-inhibited, GI) firing responses to rising glucose levels [3]. Indeed, approximately 40% of AgRP/NPY expressing neurons in the ARC and 90% of ORX neurons in the LHA demonstrate hyperpolarisation on elevation of extracellular glucose concentration [4,5]. Thus, activation of these neurons under hypoglycaemic conditions is thought to induce counter-regulatory responses, including arousal and hunger symptoms aimed at stimulating a protective feeding reaction.

Although the mechanistic underpinnings of neuronal glucose sensing remain poorly understood, it has been hypothesised that these neurons may employ a similar mechanism of detection to that seen peripherally. The low affinity hexokinase, glucokinase (GK), catalyses the phosphorylation of glucose to glucose-6-phosphate, but in pancreatic β-cells can also function as a glucose-sensor [9]. More recently, GK has also been identified within the brain and specifically in canonical glucose-sensitive nuclei, including the ARC, ventromedial nucleus and dorsomedial nucleus of the hypothalamus [10–12]. In the ARC, GK has been identified in AgRP/NPY and α-MSH/CART neurons [13]. Functional studies in hypothalamic neurons have shown that inhibition of GK function suppresses activity and/or blocks the ability of glucose to stimulate GE neurons and suppress GI neurons [14,15]. Indeed, recent studies have demonstrated the importance of hypothalamic GK in the mediation of counter-regulatory responses to insulin-induced hypoglycaemia [16]. These data support the notion that GK represents a central ‘glucostat’ capable of regulating neuronal function, and downstream protective physiological responses.

Consistent with this, we recently reported that intracerebroventricular (i.c.v) infusion of GK inhibitors such as glucosamine (GSN) [17] resulted in a rapid stimulation of protective feeding in rats [18]. Here we examine the underlying mechanism of this effect by assessing neuronal activation in chemically defined neurons induced by central GSN administration under normoglycaemic conditions.

Male Sprague–Dawley rats (Charles River) weighing 280–300 g were individually housed with *ad libitum* access to water and standard laboratory chow (Eurodent Diet, PMI Nutrition International). Animals were maintained in a light (12 h on/12 h off) and temperature controlled environment (21.5–22.5 °C). All procedures used were in accordance with the guidelines for the care and use of animals established by the UK Animals (Scientific Procedures) Act 1986.

Rats were anesthetized by intraperitoneal (i.p) administration of ketamine (100 mg/kg, National Veterinary Supplies) and xylazine (20 mg/kg, National Veterinary Supplies) and a single-guide cannula (Plastics One, VA) was inserted into the third ventricle (coordinates from bregma anteroposterior – 2.2 mm, lateral 0.9 mm, dorsoventral 8.4 mm) and cemented in place with anchoring screws, as described previously [18]. Five to eight days post surgery, *ad libitum* fed animals received either aECF (*n* = 7, CMA Microdialysis AB distributed by Linton Instrumentation) or recombinant glucosamine (GSN; CMS Chemicals 15 or 150 nmol/min, *n* = 4 and 6, respectively) via the indwelling cannula for 60 min, starting at mid light cycle at an infusion rate of 0.3 μl/min, with a priming dose of 0.9 μl/min over the first 3 min. The GSN doses used were characterized in an earlier report [18]. Food intake was measured by weighing chow pellets two hours after the termination of aECF or GSN infusion. Animals were then anesthetized with ketamine (100 mg/kg i.p) and xylazine (20 mg/kg i.p), and transcardially perfused with diethylpyrocarbonate (DEPC; Sigma)-treated 0.9% salinefollowed by phosphate-buffed 10% formalin, pH 7.0 (Sigma). Brains were removed, post-fixed in the same fixative for 4 h and then submerged overnight in 30% sucrose in DEPC-treated phosphate-buffered saline (DEPC-PBS). Brains were cut on a freezing microtome at 25 μm (1:6 series) and stored in an antifreeze solution containing 30% ethylene glycol and 20% glycerol in DEPC-PBS at −20 °C.

For quantitative assessment of neuronal activation, sectioned tissue was processed for immunohistochemical detection of cFOS immunoreactivty (FOS-IR). Each step listed below was preceded by PBS rinses for 15 min. Sections were pre-treated in 0.3% H_2_O_2_ (Sigma) for 1 h, blocked in 0.3% normal donkey serum (Sigma) in PBT (0.04% Triton X-100 (Sigma) in PBS) and then incubated with rabbit anti-FOS antibody (Calbiochem; 1:10,000) in 0.3% normal donkey serum and PBT-azide (0.02% sodium azide (Sigma) in PBT) overnight at room temperature. Sections were then incubated for 1 h with biotinylated donkey anti-rabbit serum (Jackson Laboratories; 1:500) in 0.3% normal donkey serum and PBT and then with avidin–biotin complex (ABC; Vector Elite kit; Vector laboratories; 1:250) in PBS for 1 h. The immunoperoxidase was developed in 0.04% 3,3′-diaminobenzidine tetrahydrochloride (DAB; Sigma) and 0.003% hydrogen peroxide in PBS. Sections were mounted onto polysine slides, air-dried for 30 min, counter-stained in cresyl violet (Sigma) for 1 min and dehydrated in an ascending ethanol series, before being cleared in xylene (VWR International) and cover-slipped with mounting media (Micromount, Surgipath).

Subsequent chemical identification of FOS-IR cells was achieved by dual-labelled immunofluorescence analysis. Sections were treated as described above and then incubated with goat anti-cFOS antibody (Santa Cruz, 1:1,000) and either sheep anti-α-MSH serum (Chemicon; 1:10,000), rabbit anti-ORX serum (Phoenix Pharmaceuticals; 1:10,000), or rabbit anti-MCH serum (Phoenix Pharmaceuticals; 1:10,000) in PBT overnight at room temperature. Following this, tissue was incubated with biotinylated donkey anti-goat antibody (Jackson Laboratories; 1:500) for 1 h, followed by incubation with Alexa Fluor-488 conjugated streptavidin (Molecular Probes; 1:1000) and Alexa Fluor-594 conjugated donkey anti-rabbit or anti-sheep (Molecular Probes; 1:1000) for 1 h. After mounting on polysine slides, the sections were coverslipped with anti-fade mounting medium for fluorescence (Vectashield, Vector).

To investigate the colocalisation of cFOS and NPY, a modified method combining immunofluorescence and fluorescent in situ hybridization histochemistry (FISH) was used [19]. Tissue was processed first for FOS-IR as described above using RNase-free methods. Following this, sections were rinsed thoroughly in PBS, equilibrated in 5× sodium saline citrate (SSC) for 30 min and transfered to hybridization buffer (HB) [19] for 2 h at 56 °C. A digoxgenin-labelled riboprobe (DIG-NPY) was generated from cDNA template specific to the rat NPY sequence by *in vitro* transcription with T7 polymerase, as previously described [20]. The DIG-labelled riboprobe (500 ng) was heated to 90 °C in 100 μl HB solution for 10 min, placed on ice for 5 min, and added to the tissue/HB mix and incubated for 12 to 16 hrs at 56 °C. Sections were then rinsed with 2× SSC and incubated with RNase A (Boehringer–Mannheim) in 0.5 M NaCl, 10 mM Tris–HCl, pH 8.0 and 0.5 M EDTA for 6 min at 37 °C. The sections were washed in 2× SSC for 1 h at 65 °C, and in 0.2× SSC for 1 h at 65 °C. After a brief equilibration in a solution of 0.1 M Tris–HCl, 0.1 M NaCl and 50 mM MgCl_2,_ pH 7.5 (GB1) at room temperature, the sections were transferred to blocking solution, containing 0.1 M Tris–HCl, 0.15 M NaCl and 0.5% blocking regent (PerkinElmer). Immunological detection of the DIG-NPY probe was achieved by incubating the sections in GB1 solution containing sheep anti-DIG antibody (Roche, 1:100) at room temperature overnight. The next day, following a rinse in GB1 and equilibration in 0.1 M Tris–HCl, 0.15% NaCl and 0.05% Tween 20 (TNT), DIG-NPY was visualized by Cy3 fluorophore tyramide (PerkinElmer, 1:50) for 3–10 min. Sections were briefly washed in PBS, mounted on superfrost slides, air-dried, and coverslipped with anti-fade mount medium for fluorescence (Vectashield).

Sections from all experiments were analysed and photographed with a Zeiss Axioskop 2 microscope and a Zeiss AxioCam HRc digital camera. Neuroanatomical delineation of brain regions was determined from cresyl violet counterstained sections. Immunofluorescence was observed with appropriate microscope filter sets for Alexa Fluor-488, Alexa Fluor-594 and Cy3. All data are expressed as mean ± SEM and analysed statistically using t-test, one-way ANOVA followed by Tukey's *post hoc* analysis, where appropriate, or Pearson Product Moment Correlation (SPSS version 11.5). Statistical significance was assigned at *p* < 0.05.

**Supplementary Fig. S1:**
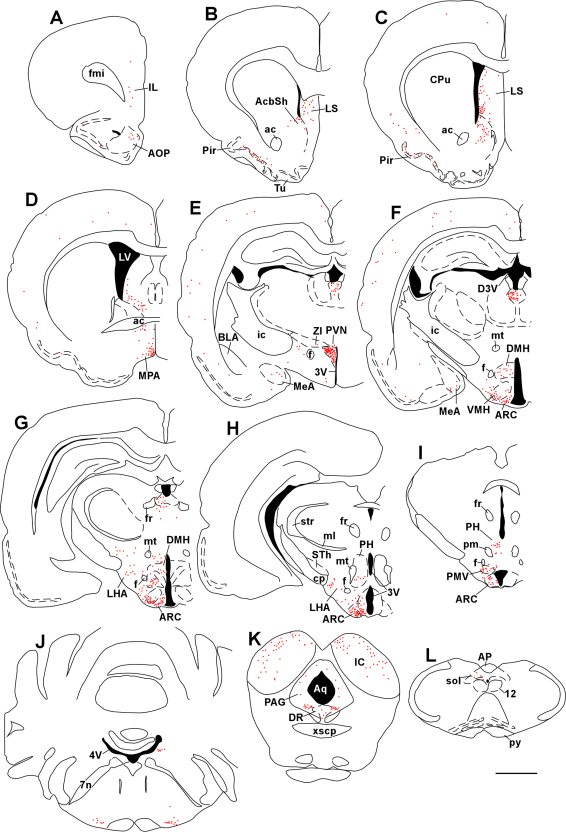
A series of low-power photomicrographs summarizing FOS-IR brain expression in a representative rat treated with glucosamine (150 nmol/min, ICV). Brain sections are an arranged in a rostral-to-caudal manner (A–L). One red dot indicates two FOS-IR cells. Scale bar in L, 1 mm. Abbreviations: 3V, third ventricle; 4V, fourth ventricle; 7n, facial nerve; 12, hypoglossal nucleus; ac, anterior commissure; AcbSh, shell portion of the accumbens nucleus; AOP, posterior part of the anterior olfactory nucleus; AP, area postrema; Aq, aqueduct; ARC, arcuate nucleus of the hypothalamus; cp, cerebral peduncle, basal part; CPu, caudate putamen; D3V, dorsal third ventricle; DMH, dorsomedial nucleus of the hypothalamus; DR, dorsal raphe nucleus; f, fornix; fmi, forceps minor of the corpus callosum; fr, fasciculus retroflexus; ic, internal capsule; IC, inferior colliculus; IL, infralimbic cortex; LHA, lateral hypothalamic area; LS, lateral septal nucleus; LSV, lateral septal nucleus, ventral part; LV, lateral ventricle; MeA, medial amygdaloid nucleus; ml, medial lemniscus; MPA, medial preoptic area; mt, mammillothalamic tract; PAG, pariaqueductal gray; PH, posterior hypothalamic area; Pir, piriform cortex; pm, principal mammillary tract; PMV, premammillary nucleus, ventral part; PVN, paraventricular nucleus of the hypothalamus; py, pyramidal tract; SO, supraoptic nucleus; sol, solitary tract; STh, subthalamic nucleus; str, superior thalamic radiation; Tu, olfactory tubercle; VMH, ventromedial nucleus of the hypothalamus; xscp, decussation of the superior cerebellar peduncle; ZI, zona incerta.

**Supplementary Fig. S2:**
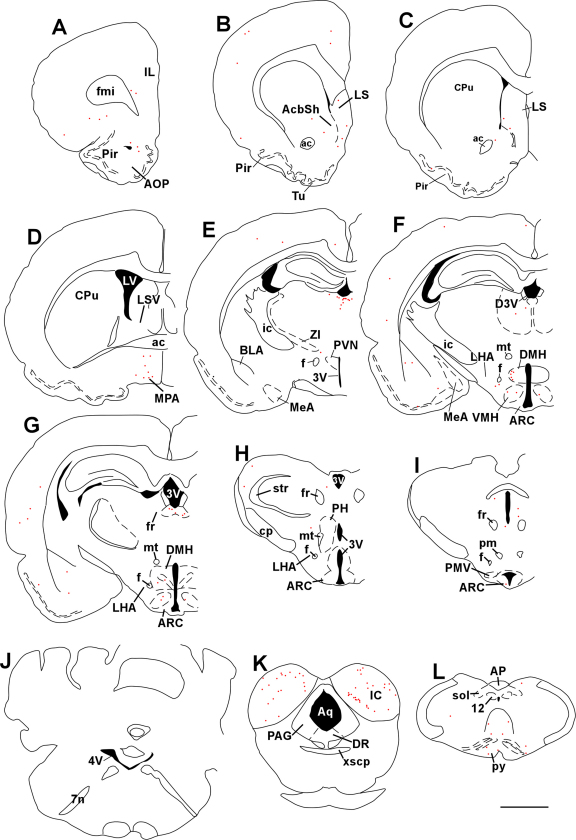
A series of low-power photomicrographs summarizing FOS-IR brain expression in a representative rat treated with ICV aECF. Brain sections are arranged in a rostral-to-caudal manner (A–L). One red dot indicates two FOS-IR cells. Scale bar in L, 1 mm. For abbreviations see Supplemental Fig. S1.

**Fig. 1:**
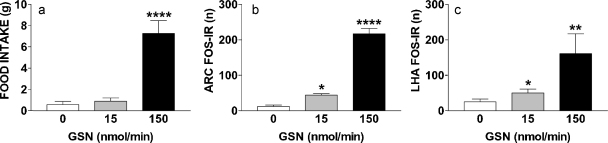
GSN significantly increased food intake and FOS-IR in the ARC and LHA. (a) GSN (150 nmol/min, i.c.v.) significantly increased 2 h food intake and FOS-IR in the (b) ARC and (c) LHA in rats compared to GSN (15 nmol/min, i.c.v.) and aECF. *p ≤ 0.05, **p ≤ 0.01, ****p ≤ 0.0001 compared to aECF.

Third ventricular infusion of the GK inhibitor GSN (150 nmol/min) rapidly and significantly stimulated feeding compared to low dose GSN (15 nmol/min) or aECF (*F*(2,10) = 32.3, *p* < 0.0001; Fig. 1A). In a directly associated manner, GSN (150 nmol/min) robustly induced FOS-IR in discrete brain regions (Supplemental Fig. S1). In particular, substantial GSN-induced FOS-IR was observed in the ARC (F(2,10) = 186.5, *p* < 0.0001; Fig. 1B) and LHA (F(2,10) = 7.4, *p* < 0.01; Fig. 1C). GSN also induced a smaller increase in FOS-IR in midline areas, particularly those related to the autonomic system and connected regions in the forebrain, such as midline thalamus, periaqueductal gray and ventral division of the bed nucleus of the stria terminalis (Supplemental Fig. S1). Infusion of aECF or the non-orexigenic dose of GSN (15 nmol/min, data not shown) induced little FOS-IR in the brain (Supplemental Fig. S2).

We further characterized the strength of the association between GSN-stimulated feeding and ARC and LHA FOS-IR induction by performing a correlation analysis. A high association was found; the greater the number of ARC and LHA neurons activated, the more effective GSN was in stimulating food intake (ARC r = 0.90, *p* < 0.001; LHA r = 0.92, *p* < 0.001).

**Fig. 2:**
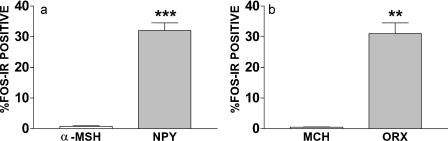
GSN significantly activates ARC NPY and LHA ORX neurons, but not ARC α-MSH or LHA MCH neurons. (a) In the ARC, GSN (150 nmol/min, i.c.v.) induced FOS-IR in less than 1% of α-MSH-IR neurons, but induced FOS-IR in approximately one-third of NPY neurons. (b) In the LHA, GSN did not increase FOS-IR in MCH-IR neurons, but produced a significant increase in FOS-IR in ORX neurons. **p ≤ 0.01, ***p ≤ 0.001.

**Fig. 3:**
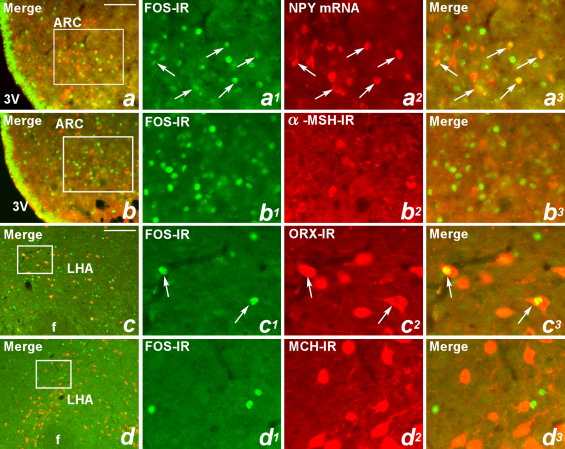
Colocalization of FOS-IR with ARC NPY mRNA and α-MSH-IR neurons and with LHA ORX-IR and MCH-IR neurons in rats treated with GSN (150 nmol/min, i.c.v.). (a–d) are merged micrographs showing representative regions of FOS-IR and neuropeptide co-expression. (a^1^–a^3^, b^1^–b^3^, c^1^–c^3^, and d^1^–d^3^) are higher-power magnification of boxes area in a–d, respectively, with a^1^, b^1^, c^1^, and d^1^ illustrating FOS-IR positive cells (green); *a*^*2*^ illustrating NPY mRNA, b^2^ illustrating α-MSH-IR, c^2^ illustrating ORX-IR and d^2^ illustrating MCH-IR (red); and a^3^, b^3^, c^3^, and d^3^ illustrating merged photographs. Arrows indicate colocolization. Scalebar in a, 75 μm, also applies to b; scalebar in c, 100 μm, also applies to d; scalebar in d^3^, 25 μm, applies to all other images. (For interpretation of the references to color in this figure legend, the reader is referred to the web version of the article.)

Dual-neurohistochemical analysis was performed to determine the chemical identity of GSN ARC and LHA activated neurons. The ARC contains two distinct populations of energy homeostasis related neurons, those containing orexigenic NPY/AgRP and those containing anorectic α-MSH. Using FISH to identify NPY mRNA expressing neurons and immunofluorescence analysis to identify FOS-IR neurons, approximately 32.0 ± 2.5% of ARC NPY/AgRP neurons were activated by 150 nmol/min GSN (Figs. 2a and 3a–a3). In contrast, using dual-label immunofluorescence on adjacent tissue from the same rats, less than 1% of ARC α-MSH neurons were activated by GSN (150 nmol/min) (Figs. 2a and 3b–b3). This resulted in a significant difference; NPY neurons expressed significantly more FOS-IR than α-MSH neurons (*t*(3) = 11.8, *p* ≤ 0.001). These data suggest that GSN significantly activates orexigenic NPY/AgRP neurons within the ARC.

The neuropeptides ORX and MCH are expressed in the LHA in distinct, non-overlapping populations. We performed dual-label immunofluorescence analysis to investigate the chemical identity of LHA GSN activated neurons. We observed that GSN (150 nmol/min) induced FOS-IR in approximately 30.7 ± 3.5% of LHA ORX neurons (Figs. 2b and 3c–c3), while in contrast saline treatment activated less than 1%. Interestingly, GSN (150 nmol/min) did not substantially activate orexigenic MCH-containing neurons; less than 1% of LHA MCH neurons co-expressed FOS-IR (Figs. 2b and 3d–d3). This difference in GSN-stimulated ORX and MCH FOS-IR induction was significant (*t*(2) = 9.0, *p* ≤ 0.01). Taken together, these data indicate that GSN-stimulated feeding is associated with ARC NPY/AgRP and LHA ORX neuronal activation.

We hypothesized that GSN would act to promote food intake by stimulating GI- neurons via a GK-dependent mechanism and predicted that this would occur via specific activation of orexigenic neurons. Using induction of the immediate-early gene cFOS as an indicator of neuronal activation [21], we observed that GSN (150 nmol/min) significantly stimulated food intake and increased neuronal activity within discrete nuclei implicated in the central control of feeding and glucose homeostasis, the ARC and LHA. Consistent with an effect on GK-mediated glucose-sensing, the pattern of hypothalamic FOS-IR activation observed was similar to that previously reported following induction of hypoglycaemia or glucopenia [22–24]. Furthermore, we also observed a strong correlation between GSN-induced hyperphagia and the degree of FOS-IR, supporting the involvement of the identified FOS-IR positive neurons in the physiological control of GK regulated food intake.

Within the ARC, we found that many neurons activated by GSN were NPY/AgRP, a population of neurons reported to express GK [13]. This pattern of activation is consistent with the observation that most (although not all) ARC neurons exhibiting suppressed calcium signalling under hypoglycaemic/glucopenic conditions were NPY positive [25,26]. Previous behavioural data also support a role for NPY neurons in the feeding response to hypoglycemia with animals genetically deficient in NPY displaying reduced glucoprivic feeding [27]. Our findings with GSN suggest that GI-NPY/AgRP neurons may use GK to detect and respond to falling glucose levels.

Within the LHA, cFOS induction by GSN was predominantly observed in ORX containing neurons, with less that 1% of MCH neurons exhibiting co-labelling. The activation of LHA ORX neurons under these conditions is consistent with previous studies demonstrating increased cFOS specifically within these neurons upon insulin-induced hypoglycaemia in rats [22,28] and electrophysiological investigation of ORX neuron excitability in hypothalamic slices [4]. Given that ORX is critical for wakefulness [29], it is possible that GSN activation of this population of neurons may be relevant to maintaining arousal, which is necessary for food seeking seek behaviour. We found no effect of GSN on MCH neuron activation, an observation corroborated by reports that MCH-containing neurons behave differently from ORX neurons, being stimulated rather than inhibited by glucose [4,7,28].

Taken together, our findings that GSN, a GK inhibitor, activates NPY and ORX expressing neurons suggests that these cells may utilise GK to detect and respond to a fall in glucose by stimulating feeding behaviour, or at very least be critical to the induction of this counter-regulatory response as a downstream component of a broader glucose sensing network. In light of the identification of GK expression within additional neuronal and non-neuronal glucose-sensing populations [3,30,31], it is at present unclear whether NPY and ORX cells types represent first-order targets of GSN action. However, our work adds to the growing body of data suggesting that brain glucose-sensing in the hypothalamus may be akin to peripheral glucose-sensing as mediated by GK.
